# Male-pattern baldness and incident coronary heart disease and risk factors in the Heinz Nixdorf Recall Study

**DOI:** 10.1371/journal.pone.0225521

**Published:** 2019-11-19

**Authors:** Sonali Pechlivanis, Stefanie Heilmann-Heimbach, Raimund Erbel, Amir A. Mahabadi, Lara M. Hochfeld, Karl-Heinz Jöckel, Markus M. Nöthen, Susanne Moebus

**Affiliations:** 1 Institute for Medical Informatics, Biometry and Epidemiology, University Hospital Essen, Essen, Germany; 2 Institute of Human Genetics, University of Bonn, Bonn, Germany; 3 Department of Genomics, Life & Brain GmbH, University of Bonn, Bonn, Germany; 4 Department of Cardiology and Vascular Medicine, West German Heart and Vascular Center, University Hospital Essen, Essen, Germany; 5 Centre for Urbane Epidemiology, University Hospital Essen, Essen, Germany; Keele University, UNITED KINGDOM

## Abstract

Male-pattern baldness (MPB) is characterized by a progressive hair loss from the frontal and vertex scalp that affects about 80% of men at the age of 80 years. Epidemiological studies show positive associations between MPB and coronary heart disease (CHD) and CHD related risk factors such as blood pressure (BP), diabetes mellitus (DM) or elevated blood lipid levels. The results however vary with regard to the pattern of hair loss (i.e. moderate, severe, frontal or vertex). Further, no study has investigated for a shared genetic determinant between MPB and CHD as well as CHD related risk factors. Using the longitudinal data from the population-based Heinz Nixdorf Recall study we aimed to systematically investigate the association between MPB and incident CHD and CHD risk factors on (i) an epidemiological (N = 1,673 males) and (ii) a genetic (N = 1,357 males) level. The prevalence of any baldness in our study population was 88% (mean age ± SD: 64±7.5 years). Compared to men with ‘no baldness’, in men with any kind of baldness a slightly increased risk for CHD (Hazard ratio [95% confidence interval (95%CI)] = 1.2 [0.8; 1.9]), a slightly higher extend of coronary artery calcification (CAC) (Beta [95%CI] = 0.2 [-0.1; 0.6]), a moderately increased risk for DM (prevalence ratio [95%CI] = 1.4 [0.9; 2.0]) and higher body mass index (BMI) (Beta [95%CI] = 0.6 [0.00003; 1.2]) seem to be indicated in the adjusted model. In contrast, the MPB genetic risk score did not show any association with CHD or CHD risk factors. Taken together, the results of our study suggest a weak association between MPB and a few CHD risk factors (CAC, DM and BMI) but do not point to MPB as a strong surrogate measure for CHD and CHD risk factors in general.

## Introduction

Male-pattern baldness (MPB) is the most common form of hair loss among men and is characterized by a progressive hair loss from the frontal and vertex scalp. The two major etiological factors are an individual’s genetic predisposition and male sex hormones. Up to 80% of men experience some degree of MPB by the age of 80 years [1].

Over the past decades, epidemiological studies have identified associations between CHD and its risk factors (e.g. metabolic syndrome, hypertension, blood cholesterol) and MPB [2–10], all suggesting that baldness may serve as an early predictive marker for CHD. However, the results are inconsistent in terms of the pattern of hair loss (i.e. moderate, severe, frontal or vertex), measured outcome types of CHD (e.g. myocardial infarction, revascularization, CHD mortality) and the reported strength of association. Moreover, no study has so far tried to systematically break down the generally observed association between MPB and CHD to specific CHD risk factors neither on an epidemiological nor at genetic level. Furthermore, the biological underpinnings of the association between MPB and CHD are so far unclear. Only speculations can be made about a potential involvement of general aging processes, hormonal pathways, metabolic factors or inflammatory processes in both traits. Therefore, the aim of our study was to systematically investigate the associations between (i) MPB and CHD and (ii) MPB and risk factors associated with CHD both at an epidemiological and at a genetic level and thereby gain insights into the associated disease mechanisms. To this end, we used the comprehensive, longitudinally assessed phenotypic and genotypic data for a subset of 1,673 male participants of the German Heinz Nixdorf Recall Study.

## Material & methods

Due to data security reasons i.e. the data contain potentially participant identifying information, the Heinz Nixdorf Recall Study does not allow sharing data as a public use file. However, for the purpose of replication, other authors/researchers are allowed to access data upon request, which is the same way the authors of the present paper obtained the data. Data requests can be addressed to: recall@uk-essen.de.

### Study population

**Fig 1:**
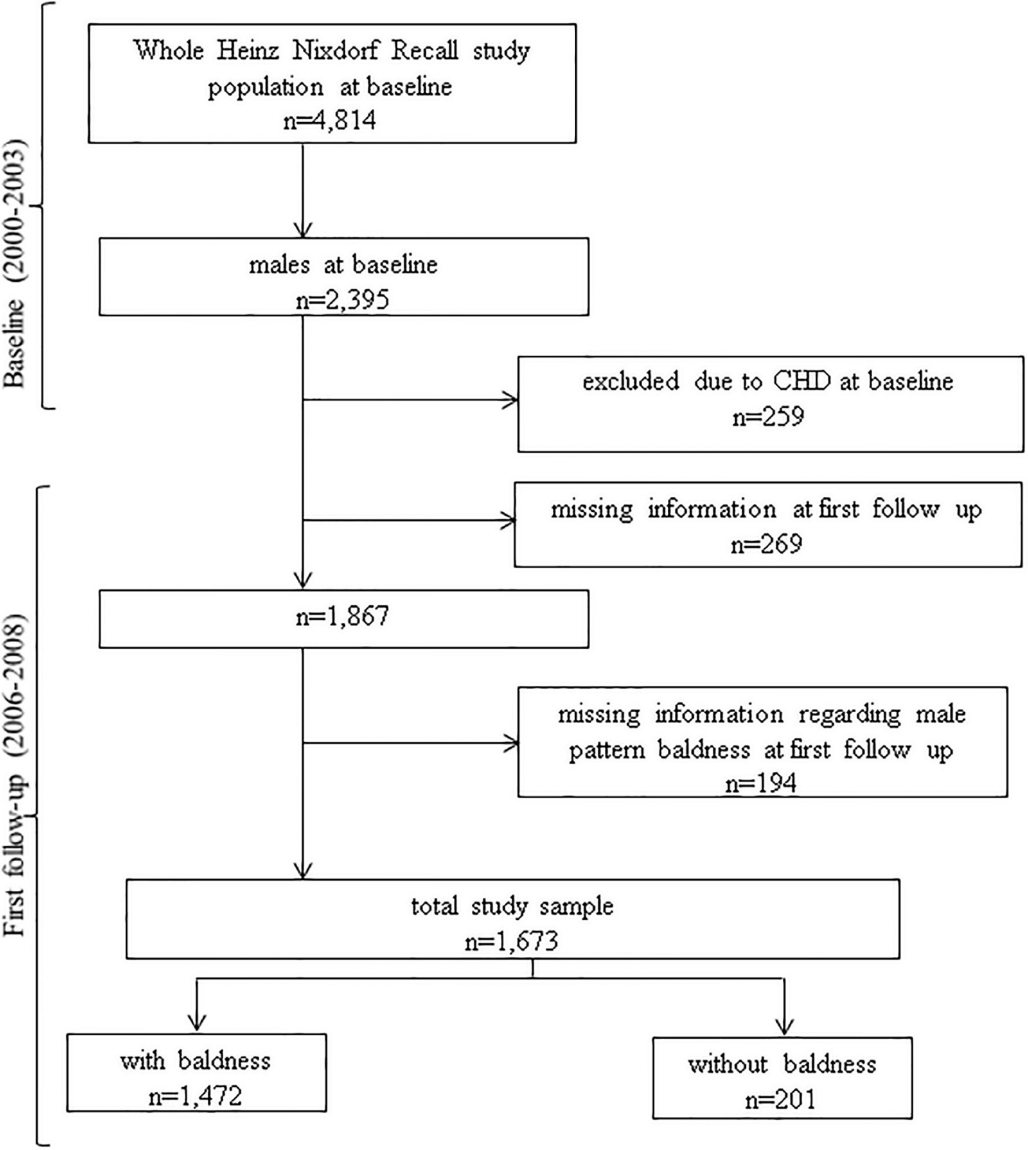
Overview of the study participant number in the Heinz Nixdorf Recall study and the present study cohort.

At baseline between December 2000 and August 2003, the Heinz Nixdorf Recall study recruited 4,814 participants (men 49.8%) from the registration lists of densely populated Ruhr metropolitan cities in Germany (residents of Essen, Bochum, and Mülheim). The participants were re-invited 5 years after the baseline examination for a second examination (2006–2008) and 10 years later for the third examination (2011–2015). The rationale and design of the study were described in detail previously [11, 12]. For the present study, we used the data from the baseline, second and third examination of 1,673 men. The analyzed study population is depicted in Fig 1. The study has been approved by the ethical committee at the University Clinic Essen, Germany and is conducted in accordance with the principles expressed in the Declaration of Helsinki. The study was certified and recertified according to DIN EN ISO 9001:2000/2008. All study participants gave their written informed consent.

### Assessment of phenotypes

#### Male-pattern baldness

Baldness patterns were assessed by dermatologists using standardized photographs from the second examination. The participants were asked to sit in front of the camera (Olympus μ800). The first photo was taken by focusing on the hairline and the receding hairlines. For the second photo, the participants were asked to bend the head slightly forward in order to show the head from above. The photographs were thereafter classified according to the Hamilton-Norwood scale (HN) [1]. For the analyses, we grouped baldness patterns into ‘no baldness’ (HN I, II, IIa) and ‘any baldness’ (HN III-VII). Moreover, we categorized sub-groups according to severity of baldness as: ‘moderate’ (HN III, IIIa, IIIvertex, IV, IVa) or ‘severe’ (HN V, Va, VI, VII) and according to localisation as ‘frontal’ (HN III, IIIa, IVa) and ‘vertex’ (HN IIIvertex, IV, V, Va, VI, VII). In total 1,657 men, aged 50–80 years, without CHD at baseline were included in this study.

#### Primary end point: Coronary heart disease

Primary end points for this study were based on clearly documented incidental CHD that met pre-defined study criteria [13]. For all the primary end points, hospital and nursing home records including ECGs, laboratory values and pathology reports were collected [14]. Death certificates and interviews with general practitioners, relatives and eyewitness were collected. Medical reports were obtained for all the reported end points [15, 16]. An external end point committee blinded for risk factor status reviewed all the documents and classified the end points at separate regular meetings twice a year. CHD was defined as myocardial infarction (fatal and non-fatal), stroke, revascularization and coronary death. In our analyses we included all incident CHD that occurred between the baseline and the third examination (n = 227, 13.6%).

#### Coronary artery calcification

Coronary artery calcification (CAC) at second examination was assessed by non-contrast-enhanced electron-beam computed tomographic scans (C-150 scanner; GE Imatron, San Francisco, Calif). Prospective ECG-triggering was done at 80% of the RR-interval. Contiguous 3-mm thick slices from the pulmonary bifurcation to the apex of the heart were obtained at an image acquisition time of 100ms. CAC was defined as a focus of at least four contiguous pixels with a computer tomography density at least 130 HU. The CAC Agatston score was computed by summing up the CAC scores of all foci in the epicardial coronary system [13]. As the distribution of CAC was right-skewed, we used a log transformation of CAC plus 1, as previously described [17].

#### Cardiovascular risk factors

Data for cardiovascular risk factors were used from the second examination phase. Medical history and smoking status (smokers (current or past) and non-smokers) were assessed by standardized computer assisted interviews [18]. Current regular use of medication, including antihypertensive or lipid lowering drugs, was recorded in a standardized medication assessment. Resting systolic (SPB) and diastolic (DBP) blood pressure was measured with the subject seated, using an automated oscillometric blood pressure device (Omron, HEM-705CP-E). The mean of the second and third value of three measurements was calculated [19]. Body mass index (BMI) was calculated as weight divided by height square (kg/m^2^). Standardized enzymatic methods were used to determine serum triglycerides, low-density lipoprotein- (LDL) and high-density lipoprotein- (HDL) cholesterol values [20]. Diabetes mellitus (DM) was defined as either of the following four criteria: (1) participants reported a history of clinically diagnosed diabetes, (2) reported intake of antidiabetic medications, (3) participants had fasting serum glucose levels (FPG) of greater than 125 mg/dL, or (4) participants had non-fasting serum glucose levels of 200 mg/dL or greater [21].

### Statistical analysis

#### Epidemiological association

For continuous data we present the mean ± standard deviation (SD) or median (first quartile: Q1, third quartile: Q3) if the distribution of data were substantially skewed. We examined the relationship between MPB and CHD using Cox proportional hazards models. The median follow-up time was 9.9±2.5year. We calculated the adjusted Hazard ratios (HR) and 95% confidence interval [95% CI]. Analysis of Kolmogorov-type supremum test (based on [22]) confirmed validity of the proportional hazards assumption. The variables used for adjustment for CHD, DM and CAC were selected a priori using directed acyclic graph (DAG) [23] as shown in S1a, S1b and S1c Fig and in S1 Table. The relationship between MPB and DM was evaluated using Poisson regression models [24, 25] to estimate the prevalence ratio (PR) and 95% CI. To study the relation between MPB and the continuous factors i.e. CAC, BMI, triglycerides, HDL-cholesterol, LDL-cholesterol, SBP and DBP we applied linear regression to calculate the Beta and 95% CI. The variables used for adjustment are shown in S1 Table. Participants with any missing data were excluded from the respective analysis. The number of missing data for each variable is presented in S2 Table. No missing data were observed for the three main variables under investigation, i.e. incident CHD, MPB and age. Missing rates for the majority of remaining outcomes such as BMI, diabetes, triglycerides, LDL-cholesterol, HDL-cholesterol, SBP and DBP were small (0.1%–0.5%). The highest missing rates were observed for CAC (missing values of 8% in “no baldness” and 12.1% “any baldness”). These missing data on CAC might lead to selection bias for the outcome CAC. The group of subjects with ‘no baldness’ was used as reference in all analyses.

#### Genetic risk score analysis

A total of 1,357 men having complete genotype and phenotype information and no CHD at baseline were selected for the genetic risk score (GRS) analysis. Blood DNA samples of these individuals were genotyped on the Illumina HumanOmni1-Quad, OmniExpress v1.0 and HumanCoreExome (v1.0 and v1.1) bead arrays. The imputation of the study participants was carried out with IMPUTE v2.3.0 [26] using the 1000 Genomes Project (release October 2014) as the reference panel [27]. The imputed data were then converted to the PLINK ped format with the threshold ≥ 0.8 using GTOOL v0.7.5. To construct an MPB GRS, 345 MPB associated genetic variants were selected from two recently published large-scale genetic analyses on MPB [28, 29]. Of the 345 published MBP risk single nucleotide polymorphism (SNPs), 215 SNPs were extracted from the HNR genetic data set. Of the remaining 130 risk SNPs, 30 SNPs were in strong linkage disequilibrium (r^2^≥0.8) with a second MPB associated variant that was included in the score, 12 SNPs were not present in the Heinz Nixdorf Recall data set and could not be replaced by a proxy SNP with r^2^≥0.8 and 88 SNPs had minor allele frequencies of <0.05 in the Heinz Nixdorf Recall study (S3A–S3E Table). To evaluate the combined effect of these MPB associated SNPs on CHD and related phenotypes, a MPB genetic risk score for each individual was constructed by summing up the number of risk alleles (0 (no risk allele) /1 (one risk allele) /2 (two risk allele)) for each of the 215 risk SNPs. The genetic risk score was calculated using the allelic scoring routine to account for the missing genotypes based on the sample allele frequency in PLINK v.19 (https://www.cog-genomics.org/plink2) [30]. The MPB genetic risk score was then analyzed per 1-unit difference in risk allele count.

The relationship between the MPB genetic risk score and CHD was estimated using an (i) age and (ii) multivariable adjusted (S1 Table) Cox proportional hazards model. To explore the relationship between the MPB genetic risk score and DM we used logistic regression to estimate the odds ratio (OR) and 95% CI (adjusted for the variables listed in S1 Table). Finally, the relationship between the MPB genetic risk score and continuous outcomes (CAC, BMI, triglycerides, HDL-cholesterol, LDL-cholesterol, SBP and DBP) was evaluated using linear regression to calculate the Beta and 95% CI. The variables used for adjustment are shown in S1 Table.

For sensitivity analyses, we used the information on family history of CHD, which is defined as fatal or non-fatal CHD or sudden cardiac death in a parent. The data was available for 1,528 (91.3%) participants. Participants who did not know whether their parents had any CHD or who did not know their biological parents (n = 145 [8.7%]) were excluded from the sensitivity analyses for CHD.

All statistical analyses were performed using SAS v.9.4 (SAS Institute, Cary, North Carolina, USA). As the goal of our analysis was the effect estimation rather than significance testing in a moderate size study cohort (N = 1,673), we decided to report point estimates and confidence intervals and to interpret our results based on the precision of these estimates instead of P-values.

## Results

### Characteristics of the study population

The basic characteristics of the study population are described in S2 Table. Of the 1,673 men of the HNR study, for whom we had documented photos of their head, only 12% (N = 201) were categorized as ‘no baldness’. According to hair loss severity the prevalence of ‘moderate baldness’ and ‘severe baldness’ was 47.9% and 40.1% respectively. Also, based on the localisation the prevalence of ‘frontal baldness’ and ‘vertex baldness’ was 8.8% and 79.2% respectively in our study group.

Participants with ‘any baldness’ were older and accordingly had higher prevalence of smoking, DM as well as in-take of lipid lowering and antihypertensive medication. Similarly, ‘any baldness’ group had lower triglycerides level, higher SBP and higher levels of CAC. In comparison to men with ‘no baldness’ (HN I, II, IIa), men with ‘any baldness’ showed a higher frequency of incident CHD (14.0% vs. 10.0%). Regarding severity and localisation of the hair loss, participants with ‘severe baldness’ and ‘vertex baldness’ were older, had lower proportions of smokers and higher amounts of CAC compared to other baldness groups. As baldness and CHD and risk factors are strongly age-dependent, the observed differences between the groups can be explained predominantly by age differences.

### Epidemiological association of MPB with CHD and CHD risk factors

#### Coronary heart disease and coronary artery calcification

**Fig 2:**
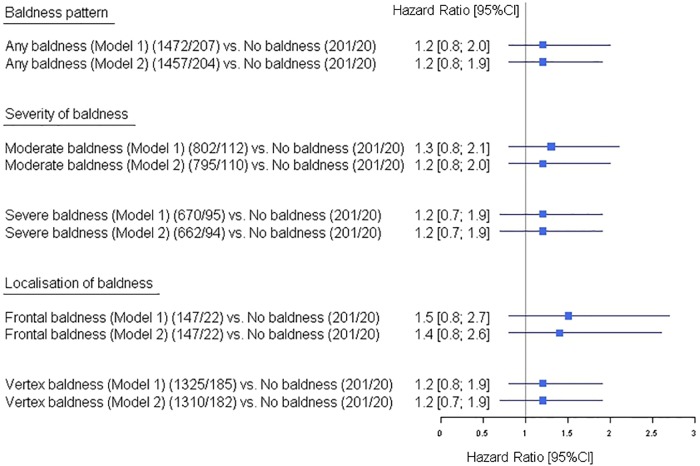
Effect of male pattern baldness as well as the baldness sub-groups based on severity and localisation on incident coronary heart disease in the Heinz Nixdorf Recall study. Models: the adjustment sets for outcome CHD are indicated in the S1 Table 95%CI: 95% confidence interval. The numbers are given as total number of participants/number of events.

Our analysis for an association between incident CHD and MPB revealed a moderately increased risk for CHD events in men with ‘any baldness’ (Model 1, HR [95%CI] = 1.2 [0.8; 2.0]). The analyses based on baldness severity (‘moderate’, ‘severe’) and localisation (‘frontal’, ‘vertex’) revealed similar elevated risk estimates and 95% CIs (Fig 2). All reported HRs remained unchanged after adjustment for potential CHD risk factors. As sensitivity analyses, we additionally adjusted model 1 for family history of CHD. The effect size (HR [95%CI] = 1.1 [0.7; 1.8]) for the association with CHD in men with ‘any baldness’ remained similar to the analyses without information on family history (data not shown).

The results of the association between MPB and CAC in our study, showed increased log (CAC+1) values in all the baldness subgroups compared to ‘no baldness’ (Model 2, Beta [95%CI] = 0.2 [-0.1; 0.6]) (Table 1).

**Table 1:** Association between male pattern baldness and risk factors for coronary heart disease.

Phenotype (N)	No baldness N	Any baldness N	Severity of baldness		Localisation of baldness	
			Moderate baldness N	Severe baldness N	Frontal baldness N	Vertex baldness N
**CAC (1479)**	**185**	**1294**	**703**	**591**	**128**	**1166**
Model 1: Beta [95%CI]	-	0.3 [-0.03; 0.7]	0.3 [-0.1; 0.7]	0.4 [-0.01; 0.8]	0.2 [-0.3; 0.7]	0.3 [-0.02; 0.7]
Model 2: Beta [95%CI]	-	0.2 [-0.1; 0.6]	0.2 [-0.2; 0.5]	0.2 [-0.1; 0.6]	0.1 [-0.4; 0.6]	0.2 [-0.1; 0.6]
**BMI (1667)**	**201**	**1466**	**799**	**667**	**147**	**1319**
Model 1: Beta [95%CI]	-	0.6 [0.00003; 1.2]	0.6 [-0.06; 1.2]	0.7 [-0.002; 1.3]	0.3 [-0.6; 1.2]	0.6 [0.03; 1.3]
**HDL-cholesterol (1666)**	**201**	**1465**	**799**	**666**	**147**	**1318**
Model 1: Beta [95%CI]	-	-1.8 [-3.8; 0.2]	-1.9 [-4.0; 0.1]	-1.6 [-3.7; 0.6]	-0.7 [-3.6; 2.1]	-1.9 [-3.9; 0.1]
Model 2: Beta [95%CI]	-	-1.8 [-3.7; 0.2]	-1.9 [-4.0; 0.2]	-1.6 [-3.8;-0.5]	-0.7 [-3.6; 2.1]	-1.9 [-3.9; 0.1]
**LDL-cholesterol (1666)**	**201**	**1465**	**799**	**666**	**147**	**1318**
Model 1: Beta [95%CI]	-	-0.05 [-5.1; 4.9]	-1.4 [-6.6; 3.8]	1.8 [-3.6; 7.2]	-1.7 [-8.8; 5.5]	0.1 [-4.9; 5.2]
Model 2: Beta [95%CI]	-	0.3 [-4.7; 5.2]	-1.1 [-6.2; 3.9]	1.3 [-3.9; 6.5]	-1.5 [-8.6; 5.6]	0.5 [-4.5; 5.5]
**Triglycerides (1666)**	**201**	**1465**	**799**	**666**	**147**	**1318**
Model 1: Beta [95%CI]	-	2.8 [-10.5; 16.2]	2.8 [-11.2; 17.3]	2.9 [-11.5; 17.3]	-4.4 [-23.5;1 4.7]	3.7 [-9.7; 17.2]
Model 2: Beta [95%CI]	-	2.9 [-10.4; 16.2]	2.5 [-11.4; 16.3]	3.5 [-10.8; 17.8]	-4.2 [-23.2; 14.7]	3.8 [-9.6; 17.2]
**SBP (1671)**	**201**	**1470**	**801**	**669**	**147**	**1323**
Model 1: Beta [95%CI]	-	1.4 [-1.4; 4.1]	1.3 [-1.6; 4.2]	1.4 [-1.6; 4.4]	0.8 [-3.2; 4.8]	1.4 [-1.4; 4.2]
Model 2: Beta [95%CI]	-	1.2 [-1.5; 4.0]	1.2 [-1.7; 4.1]	1.3 [-1.7; 4.3]	0.7 [-3.3; 4.7]	1.3 [-1.5; 4.1]
**DBP (1671)**	**201**	**1470**	**801**	**669**	**147**	**1323**
Model 1: Beta [95%CI]	-	0.3 [-1.3; 1.8]	0.3 [-1.3; 1.9]	0.2 [-1.5; 1.9]	-0.2 [-2.4; 2.1]	0.3 [-1.2; 1.9]
Model 2: Beta [95%CI]	-	0.3 [-1.3; 1.8]	0.3 [-1.3; 1.9]	0.2 [-1.5; 1.9]	-0.2 [-2.4; 2.1]	0.3 [-1.2; 1.9]

CAC: coronary artery calcification, BMI: body mass index, HDL: high density lipoprotein, LDL: low density lipoprotein, SBP: systolic blood pressure, DBP: diastolic blood pressure, [95%CI]: 95% confidence interval. No baldness was used as the reference group. Models: the adjustment sets for each phenotype are indicated in the S1 Table.

#### Diabetes mellitus and body mass index

**Fig 3:**
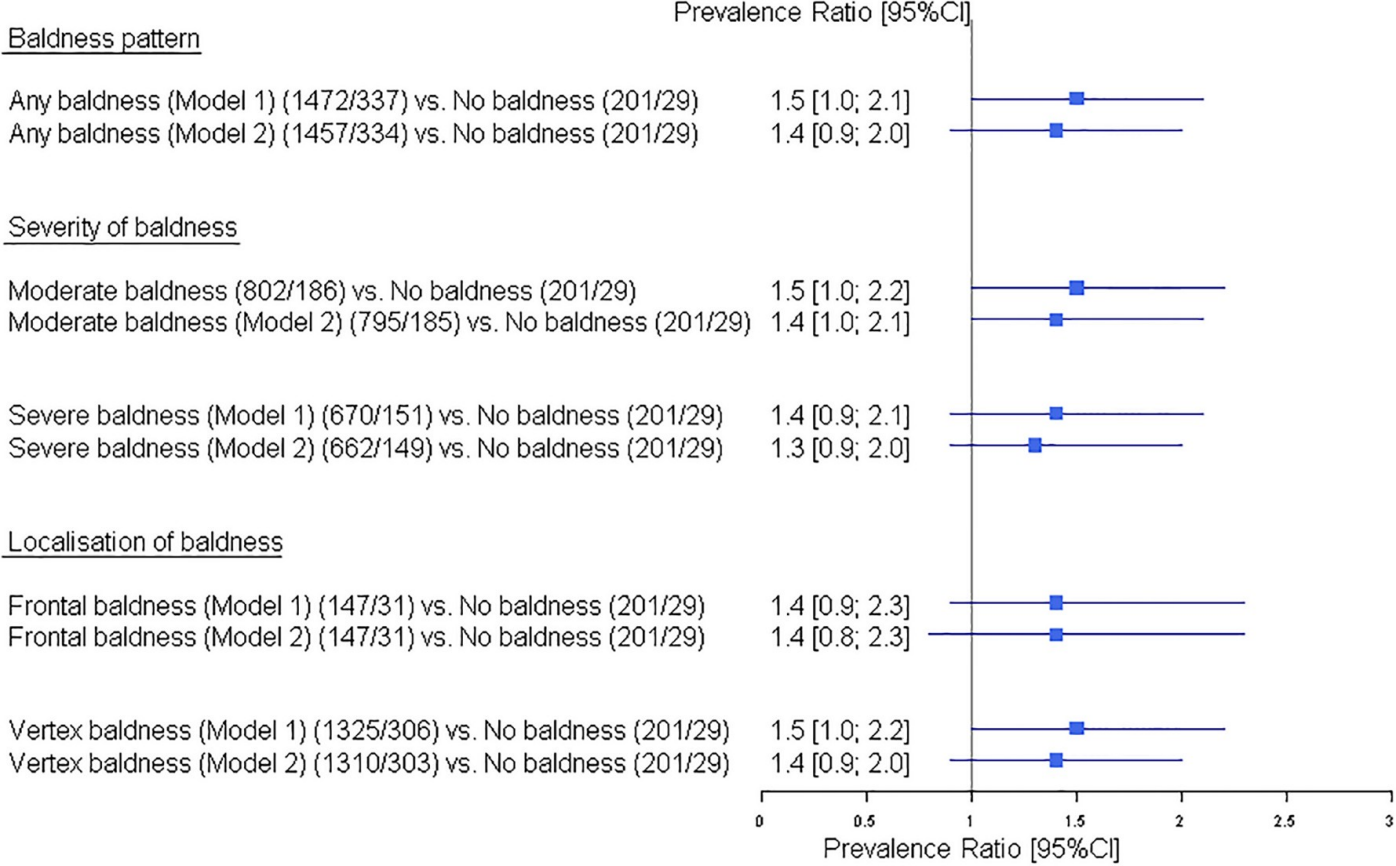
Effect of male pattern baldness as well as the baldness sub-groups based on severity and localisation on diabetes mellitus in the Heinz Nixdorf Recall study. Models: the adjustment sets for outcome diabetes mellitus are indicated in the S1 Table. 95%CI: 95% confidence interval. The numbers are given as total number of participants/number of participants with diabetes mellitus.

Regarding DM and BMI, the following observation were made: men with ‘any baldness’ had a moderately increased risk of DM compared to men with ‘no baldness’ (PR [95%CI] = 1.5 [1.0; 2.1]), without any change after full adjustment (Model 2, Fig 3). Risk estimates in the subgroups of baldness are of the same magnitude (i.e. ‘moderate baldness’ (PR [95%CI] = 1.5 [1.0; 2.2]), ‘severe baldness’ PR [95%CI] = 1.4 [0.9; 2.1], ‘frontal baldness’ PR [95%CI] = 1.4 [0.9; 2.3] and ‘vertex baldness’ PR [95%CI] = 1.5 [1.0; 2.2]), again without changes after full adjustments (Model 2, Fig 3).

Similarly, we observed weak positive associations with BMI in men with ‘any baldness’ (Beta [95%CI] = 0.6 [0.00003; 1.2]) and in all their subgroups (i.e. ‘severe baldness’ Beta [95%CI] = 0.7 [-0.002; 1.3]), ‘vertex baldness’ Beta [95%CI] = 0.6 [0.03; 1.3], ‘moderate baldness’ Beta [95%CI] = 0.6 [-0.06; 1.2] and ‘frontal baldness’ Beta [95%CI] = 0.3 [-0.6; 1.2]) when compared to men with ‘no baldness’ (Table 1).

#### Lipid levels and blood pressure

The analyses for an epidemiological association between lipid traits (HDL-cholesterol, LDL-cholesterol, triglycerides) and MPB revealed that men with ‘any baldness’ tended to have lower HDL-cholesterol (Model 2, Beta [95%CI] = -1.8 [-3.7; 0.2]), higher LDL-cholesterol (Model 2, Beta [95%CI] = 0.3 [-4.7; 5.2]) and higher triglyceride levels (Model 2, Beta [95%CI] = 2.9 [-10.4; 16.2]). While lower HDL-cholesterol levels were observed in all subgroups of baldness when compared to men with ‘no baldness’, the results were inconsistent regarding LDL-cholesterol and triglyceride levels (Model 2, Table 1).

Moreover, we performed two separate analyses to examine the potential association between MPB and SBP and DPB. Here, men with ‘any baldness’ (Model 2, Beta [95%CI] = 1.2 [-1.5; 4.0]) and all the subgroups (Table 1) had higher SBP compared to the men with ‘no baldness’ (Table 1). Similarly for DBP, men with ‘any baldness’ (Model 2, Beta [95%CI] = 0.3 [-1.3; 1.8]) and it’s subgroups except the ‘frontal baldness’ (Table 1) showed higher DBP levels when compared to men with ‘no baldness’. While there was a general tendency towards higher LDL-cholesterol, triglycerides, SBP and DBP levels and lower HDL-cholesterol in men with baldness compared to men with ‘no baldness’, the observed confidence intervals were large.

#### Association of the MPB genetic risk score with CHD and related phenotypes

To identify a potential genetic overlap between MPB and CHD or risk factors for CHD, we next sought to investigate for an association between the MPB genetic risk score consisting of 215 MPB associated risk SNPs from two recent large-scale genetic studies on MPB. The established MPB genetic risk score did not show any association with CHD (Model 1, Table 2) (HR [95%CI] = 1.00 [0.99; 1.01]) or the CHD risk factors (DM (OR [95%CI] = 1.00 [0.98; 1.01]), CAC (Beta [95%CI] = 0.007 [-0.01; 0.02]), BMI (Beta [95%CI] = 0.002 [-0.02; 0.02]), HDL-cholesterol (Beta [95%CI] = -0.01 [-0.07; 0.06]), LDL-cholesterol (Beta [95%CI] = 0.09 [-0.07; 0.26]), triglyceride (Beta [95%CI] = -0.08 [-0.52; 0.37]), SBP (Beta [95%CI] = -0.03 [-0.12; 0.06]) and DBP (Beta [95%CI] = -0.02 [-0.07; 0.03])] as well as in full adjusted model (Model 2, Table 2).

**Table 2:** Association of the unweighted male pattern baldness genetic risk score with incident coronary heart disease (CHD) and risk factors for CHD.

Phenotype	Unweighted MPB genetic risk score
**CHD, N (Events)**	**1169 (188)**
HR [95%CI], Model 1	1.00 [0.99; 1.01]
HR [95%CI], Model 2	1.00 [0.99; 1.01]
HR [95%CI], Model 3	1.00 [0.98; 1.01]
**Diabetes mellitus, N (Cases)**	**1069 (288)**
OR [95%CI], Model 1	1.00 [0.98; 1.01]
OR [95%CI], Model 2	1.00 [0.98; 1.01]
**CAC, N**	**1204**
Beta [95%CI], Model 1	0.007[-0.01; 0.02]
Beta [95%CI], Model 2	0.007 [-0.004; 0.02]
**BMI, N**	**1357**
Beta [95%CI], Model 1	0.002 [-0.02; 0.02]
**HDL-cholesterol, N**	**1357**
Beta [95%CI], Model 1	-0.01 [-0.07; 0.06]
Beta [95%CI], Model 2	-0.01 [-0.08; 0.05]
**LDL-cholesterol, N**	**1357**
Beta [95%CI], Model 1	0.09 [-0.07; 0.26]
Beta [95%CI], Model 2	0.05 [-0.10; 0.21]
**Triglycerides, N**	**1357**
Beta [95%CI], Model 1	-0.08 [-0.52; 0.37]
Beta [95%CI], Model 2	-0.03 [-0.48; 0.41]
**Systolic blood pressure, N**	**1357**
Beta [95%CI], Model 1	-0.03 [-0.12; 0.06]
Beta [95%CI], Model 2	-0.04 [-0.13; 0.05]
**Diastolic blood pressure, N**	**1357**
Beta [95%CI], Model 1	-0.02 [-0.07; 0.03]
Beta [95%CI], Model 2	-0.02 [-0.07; 0.03]

CHD: coronary heart disease, HR: hazard ratio, [95%CI]: 95%confidence interval, OR: odds ratio, CAC: coronary artery calcification, BMI: body mass index, HDL: high density lipoprotein, LDL: low density lipoprotein, SBP: systolic blood pressure, DBP: diastolic blood pressure. Models: the adjustment sets for each phenotype are indicated in the S1 Table.

## Discussion & conclusion

In our present study, we systematically investigated the association between MPB with (i) CHD and (ii) risk factors for CHD both on an epidemiological and at a genetic level using the data of the HNR Study. While we did not detect any genetic associations between MPB and CHD or related phenotypes, our epidemiological data point towards an increased risk for CHD in men with baldness. In contrast to previously published findings by Yamada et al. (2013) and Trieu et al. (2014) who reported a dose-response relation between CHD and MPB, where vertex pattern and greater severity of MPB resulted in increased CHD-risk [10, 31], our data suggest increased risk in MPB in men with frontal (HN III, IIIa, IVa) and moderate (HN III, IIIa, IIIvertex, IV, IVa) baldness. Our sensitivity analyses adjusting for family history of CHD did not alter our results for the association between MPB and CHD. The effect sizes from our study are similar to the other published studies [5, 10, 31, 32], that reported increased risk for CHD in Trieu et al. (2014) (OR [95%CI]: 1.22 [1.07–1.39]), Lotufo et al. (2000) (RR [95%CI]): ‘frontal baldness’ (1.09 [0.99–1.27]), ‘mild vertex baldness’ (1.24 [1.05–1.46]), ‘moderate vertex baldness’ (1.33 [1.08–1.62]) and ‘severe vertex baldness’ (1.30 [1.03–1.63])), Shahar et al.(2008) (OR [95%CI]): ‘frontal baldness’ (1.28 [0.97–1.68]), ‘mild vertex baldness’ (1.02 [0.8–1.30]), ‘moderate vertex baldness’ (1.40 [1.05–1.86]) and ‘severe vertex baldness’ (1.18 [0.93–1.49])) and Yamada et al. (2013) (RR [95%CI]): ‘severe vertex baldness’ (1.48 [1.04–2.11]), ‘mild vertex baldness’ (1.18 [1.04–1.35]), ‘moderate vertex baldness’ (1.36 [1.16–1.58]) and ‘frontal baldness’ (1.11 [0.92–1.32])). The study from Herrera et al. (1995) reported that the extend of baldness was not associated with CHD mortality, however, the amount of progression of baldness was associated with CHD mortality (RR [95%CI]; moderate progression (1.7 [1.0–2.8]) and rapid progression (3.8 [1.9–7.7])) [33]. To better understand the pathophysiology of CHD, Sari et al. (2015) investigated the association between MPB and angiographic CAD severity and collateral development, concluding no relation between them [34]. Moreover, our data indicated an increase in log(CAC+1) values in all baldness subgroups compared to ‘no baldness’. This is one of the first studies looking at the association between MPB and CAC. The result of our study suggests that MPB could be considered as an additional risk for subclinical atherosclerosis. This is in line with the study by Dogramaci et al. (2009), where the authors reported an association between severe vertex baldness and intima-media thickness [35].

Regarding other CHD associated risk factors, our data revealed higher point estimates for DM and BMI in men with MPB. These findings are in line with previous studies that reported a positive association between MPB and DM and insulin-linked disturbances in the Finish population [36] and MPB and increased risk of mortality from DM and heart disease in Taiwanese [9]. The study by Hirsso et al. (2007) and a meta-analysis by Trieu et al. (2014), also found a positive associations between MPB and BMI [31, 37]. The association results regarding MPB and lipid as well as blood traits were inconsistent across baldness categories in our study, however men with ‘any baldness’ tended to have lower HDL-cholesterol, higher fasting triglyceride, SBP and DBP levels than men without baldness. These results are consistent with the results from Trieu et al. (2014) that found similar non-significant associations between MPB and higher fasting triglyceride and lower HDL cholesterol blood levels [31]. Another study by Sharma et al. (2013) found significantly increased blood pressure, HDL-cholesterol, LDL-cholesterol, serum lipoprotein-a, serum homocysteine and serum adiponectin levels in MPB cases compared to controls [38].

The strengths of the present study was its longitudinal design with longer follow-up time (9.9±2.5 years), stringent predefined end point criteria, and availability of a wide range of CHD risk factors as well as genetic data. In the longitudinal or cross-sectional analyses in the present study, the experienced dermatologists who assessed the participant’s hair status based on standardized photographs were not aware of the individual results for any other outcome measurements. Hence, information bias would have no influence on the study results. Possible confounders like age; which is not only an important risk factor for CHD but is also related to the balding process; as well as other CHD risk factors did not play a role in our study. We have adjusted our analyses for age as well as other risk factors. The results of the age as well as risk factors adjusted models were similar. The sensitivity analyses adjusting for family history of CHD did not alter our results for the association between MPB and CHD. Hence, the different distributions of unmeasured confounders are unlikely to have affected our findings. Few participants (n = 194) had missing value of CAC. For the outcome CAC, these missing data might have led to selection bias for the association between MPB and CAC. The biggest limitation of this study was its smaller sample size, which has restricted us to detect small effect sizes with precision. However, the effect sizes as well as 95% CI from our study were comparable to other larger studies [5, 10, 31, 32].

Looking at our data, ‘any baldness’ is a normal age-related process as 88% of our study population has any type of baldness. One could also interpret the results of our study as having no baldness or having hair is good for our health. Since it is difficult to compare the protective effects of ‘no baldness’ with other published studies which used baldness as a risk factor, we decided to use ‘no baldness’ as reference group so that our results could be compared with other studies. In summary, the results of our study suggest a weak association between MPB and CHD and a few CHD risk factors but do not point to MPB as a strong surrogate measure for CHD and CHD risk factors in general. Although, the associations are weak, one can still speculate that the associations between CHD and MPB may be driven by the cumulative effect of shared etiological components between MPB and different CHD risk factors such as DM, BMI and CAC. Large-scale prospective studies are warranted to confirm this hypothesis. Furthermore, our genetic risk score analysis that tested for a summatory effect of MPB associated variants on an individual’s risk for CHD and CHD risk factors did not yield any supportive evidence for a shared genetic basis between MPB and these traits. These data are in line with previously published data from an LD-score based analysis that did not find any genetic correlation between MPB and CHD associated traits [19]. While the GRS and LD-score regression methods are designed to identify a general shared genetic component between traits, these methods are not aiming at identifying shared individual pathways or even genetic risk factors at single loci. These individual shared biological factors may however affect disease risk for both CHD and MPB and contribute to the epidemiological association between the traits. Further studies, in large and phenotypically as well as genetically well-characterized cohorts are needed to enable the detailed investigation of such overlapping associations at single loci and pathways. If the results of our study are confirmed in larger studies, baldness may be worth considering as an early prognostic marker of CHD and atherosclerosis. Additional studies will then be necessary to test for the clinical utility of including MPB into current risk models for CHD and associated phenotypes. Together with additional analyses that focus on the investigation of the underlying biological causes of the association, this may eventually have implications for prevention, diagnosis and treatment of both, CHD and MPB.

## Supporting information

S1 Fig **A) Directed acyclic graph (DAG) on the hypothesized associations between coronary heart disease, male pattern baldness and covariates in our study. B) DAG on the hypothesized associations between diabetes mellitus, male pattern baldness and covariates in our study**. **C) DAG on the hypothesized associations between coronary artery calcification, male pattern baldness and covariates in our study**. Source: Created with DAGitty (www.dagitty.net, Textor et al. 2011).
(TIF)

S1 Table Adjustment set used for each of the phenotypes. (DOCX)

S2 Table Basic characteristic of the study population. (DOCX)

S3 Table A) Excluded genetic variants due to linkage disequilibrium (R^2^≥0.80). B) Excluded common genetic variants between the Heilmann-Heimbach et al. and Hagenaars et al. C) Excluded genetic variants as no proxy was found. D) Excluded genetic variants as R^2^≤0.8 with the proxies. E) Excluded 88 genetic variants with minor allele frequency < 0.05.
(DOCX)
