# Supplementary figures and images for: Male-pattern baldness and incident coronary heart disease and risk factors in the Heinz Nixdorf Recall Study
Source: PLoS One. 2019 Nov 19;14(11):e0225521. doi: 10.1371/journal.pone.0225521 (PMC6863534; doi:10.1371/journal.pone.0225521)

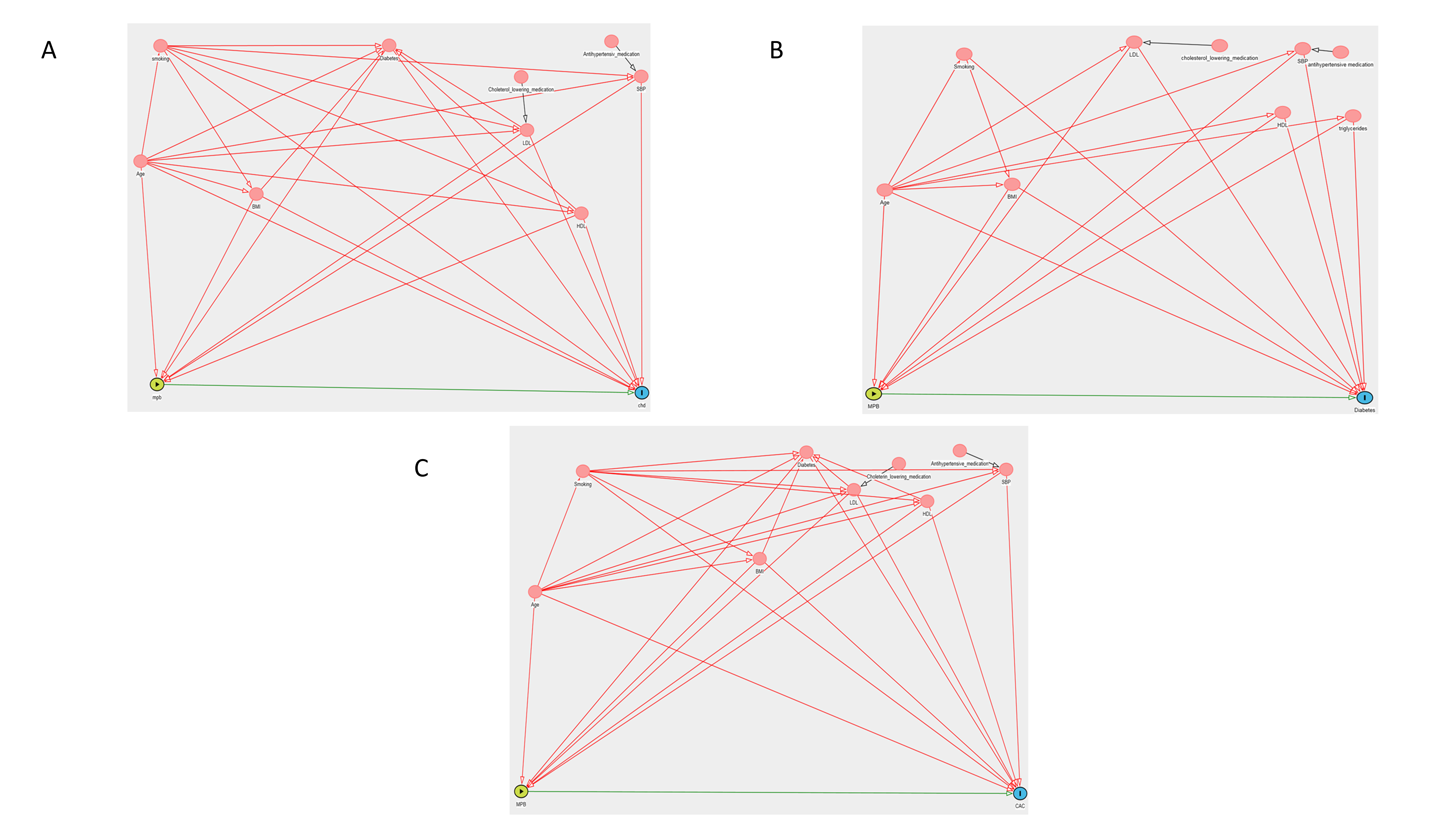

Supplement: S1 Fig — A) Directed acyclic graph (DAG) on the hypothesized associations between coronary heart disease, male pattern baldness and covariates in our study. B) DAG on the hypothesized associations between diabetes mellitus, male pattern baldness and covariates in our study. C) DAG on the hypothesized associations between coronary artery calcification, male pattern baldness and covariates in our study. Source: Created with DAGitty (www.dagitty.net, Textor et al. 2011). (TIF) [file pone.0225521.s001.tif]
