# Supplementary material for: Male-pattern baldness and incident coronary heart disease and risk factors in the Heinz Nixdorf Recall Study
Source: PLoS One. 2019 Nov 19;14(11):e0225521. doi: 10.1371/journal.pone.0225521 (PMC6863534; doi:10.1371/journal.pone.0225521)
Supplement: S1 Table — (DOCX) [file pone.0225521.s002.docx]

**S1 Table: Adjustment set used for each of the phenotypes**

| Phenotypes | Model 1 | Model 2 |
| --- | --- | --- |
| CHD | Age | Model 1 + BMI, HDL-cholesterol, LDL-cholesterol, SBP, DM |
| Diabetes mellitus | Age | Model 1 + BMI, HDL-cholesterol, LDL-cholesterol, SBP |
| CAC | Age | Model 1 + BMI, DM, HDL-cholesterol, LDL-cholesterol, SBP |
| BMI | Age |  |
| Triglycerides | Age and fasting status | Model 1 + use of a cholesterol lowering medication |
| HDL-cholesterol | Age | Model 1 + use of a cholesterol lowering medication |
| LDL-cholesterol | Age | Model 1 + use of a cholesterol lowering medication |
| SBP | Age | Model 1 + use of antihypertensive medication |
| DBP | Age | Model 1 + use of antihypertensive medication |

CHD: coronary heart disease, CAC: coronary artery calcification, BMI: body mass index, HDL: high density lipoprotein, LDL: low density lipoprotein, SBP: systolic blood pressure, DBP: diastolic blood pressure. CAC was log transformed using log(CAC+1)
