# Supplementary material for: Male-pattern baldness and incident coronary heart disease and risk factors in the Heinz Nixdorf Recall Study
Source: PLoS One. 2019 Nov 19;14(11):e0225521. doi: 10.1371/journal.pone.0225521 (PMC6863534; doi:10.1371/journal.pone.0225521)
Supplement: S2 Table — (DOCX) [file pone.0225521.s003.docx]

**S2 Table: Basic characteristic of the study population.**

| Characteristic | No baldness | Any baldness | Moderate baldness | Severe baldness | Frontal baldness | Vertex baldness |
| --- | --- | --- | --- | --- | --- | --- |
| N (%) | 201 (12.0) | 1472 (88.0) | 802 (47.9) | 670 (40.1) | 147 (8.8) | 1325 (79.2) |
| Age (years) | 60.9±6.9 | 64.4±7.5 | 63.4±7.2 | 65.6±7.5 | 62.4±7.5 | 64.6±7.4 |
| CHD | 20 (10.0) | 207 (14.0) | 112 (13.9) | 95 (14.2) | 22 (15.0) | 185 (14.0) |
| log(CAC+1); median (Q1; Q3) | 3.7 (0.8; 5.5) | 4.7 (2.5; 6.0) | 4.5 (2.1; 6.0) | 4.9 (2.9; 6.1) | 3.8 (0; 6.0) | 4.2 (2.6; 6.0) |
| Missing N (%) | 16 (8.0) | 178 (12.1) | 99 (12.3) | 79 (11.8) | 19 (12.9) | 159 (12.0) |
| BMI (kg/m^2^) | 27.8±4.5 | 28.5±4.1 | 28.5±4.1 | 28.6±4.0 | 28.2±3.9 | 28.5±4.1 |
| Missing N (%) | - | 6 (0.4) | 3 (0.4) | 3 (0.4) | - | 6 (0.5) |
| HDL-cholesterol (mg/dl) | 54.7±13.9 | 53.3±13.3 | 53.0±13.1 | 53.7±13.5 | 54.1±12.4 | 53.2±13.4 |
| Missing N (%) | - | 7 (0.5) | 3 (0.4) | 4 (0.6) | - | 7 (0.5) |
| LDL-cholesterol (mg/dl) | 131.2±34.10 | 130.5±33.4 | 129.4±34.3 | 131.9±32.3 | 129.2±34.3 | 130.7±33.3 |
| Missing N (%) | - | 7 (0.5) | 3 (0.4) | 4 (0.6) | - | 7 (0.5) |
| Triglycerides (mg/dl) median  (Q1; Q3) | 131.0  (86.0; 177.0) | 121.0  (87.0; 172.0) | 123.0  (87.0; 175.0) | 119.0  (86.0; 171.0) | 115.0  (86.0; 160.0) | 122.0  (87.0; 174.0) |
| Missing N (%) | - | 7 (0.5) | 3 (0.4) | 4 (0.6) | - | 7 (0.5) |
| SBP (mmHg) | 135.8 ±18.4 | 138.8±19.1 | 138.3±19.0 | 139.4±19.3 | 137.2±17.7 | 139.0±19.3 |
| Missing N (%) | - | 2 (0.1) | 1 (0.1) | 1 (0.1) | - | 2 (0.2) |
| DBP (mmHg) | 82.0±10.7 | 81.5±10.5 | 81.8±10.5 | 81.2±10.6 | 81.5±10.6 | 81.5±10.5 |
| Missing N (%) | - | 2 (0.1) | 1 (0.1) | 1 (0.1) | - | 2 (0.2) |
| Diabetes mellitus | 29 (14.4) | 337 (22.9) | 186 (23.2) | 151 (22.5) | 31 (21.1) | 306 (23.1) |
| Lipid lowering medication | 42 (20.9) | 341 (23.2) | 192 (23.9) | 149 (22.2) | 30 (20.4) | 311 (23.5) |
| Antihypertensive medication | 79 (39.3) | 713 (48.4) | 381 (47.5) | 332 (49.6) | 64 (43.5) | 649 (49.0) |
| Smoker (current or past) | 133 (66.2) | 1051 (71.6) | 582 (72.8) | 469(70.1) | 114 (77.6) | 937 (70.9) |

Data are given as mean ± SD or n (percentage), unless otherwise indicated. Missing indicates the number of participants with missing data for a particular phenotype. CHD: coronary heart disease, CAC: coronary artery calcification, BMI: body mass index, HDL: high density lipoprotein, LDL: low density lipoprotein, SBP: systolic blood pressure, DBP: diastolic blood pressure.
