# Supplementary material for: Male-pattern baldness and incident coronary heart disease and risk factors in the Heinz Nixdorf Recall Study
Source: PLoS One. 2019 Nov 19;14(11):e0225521. doi: 10.1371/journal.pone.0225521 (PMC6863534; doi:10.1371/journal.pone.0225521)
Supplement: S3 Table — A) Excluded genetic variants due to linkage disequilibrium (R2≥0.80). B) Excluded common genetic variants between the Heilmann-Heimbach et al. and Hagenaars et al. C) Excluded genetic variants as no proxy was found. D) Excluded genetic variants as R2≤0.8 with the proxies. E) Excluded 88 genetic variants with minor allele frequency < 0.05. (DOCX) [file pone.0225521.s004.docx]

**S3A Table. Excluded genetic variants due to linkage disequilibrium (r^2^≥0.80)**

| Excluded genetic variants | Included genetic variants |
| --- | --- |
| rs6683032 | rs17371253 |
| rs17186024 | rs17185996 |
| rs10919382 | rs2421326 |
| rs2143109 | rs2206310 |
| rs6752754 | rs12997617 |
| rs7349332 | rs74333950 |
| rs9878451 | rs9850626 |
| rs13060333 | rs4679956 |
| rs7680591 | rs982804 |
| rs1422798 | rs17643057 |
| rs79032435 | rs9380830 |
| rs12702266 | rs12702262 |
| rs79206101 | rs118111611 |
| rs1907350 | rs1907352 |
| rs3781452 | rs3781458 |
| rs7974900 | rs9300169 |
| rs11049231 | rs10843026 |
| rs76972608 | rs78472887 |
| rs2117234 | rs7182742 |
| rs246180 | rs246185 |
| rs8085664 | rs12606816 |
| rs2242288 | rs59304342 |
| rs77775907 | rs113923480 |
| rs77340799 (proxy for rs112348497) | rs75732647 |
| rs11659559 | rs17594358 |
| rs11659559 | rs8095770 |
| rs77767830 | rs117769774 |
| rs116315504 | rs115282290 |
| rs6113492 | rs201593 |
| rs7280071 | rs68088846 |

**S3B Table: Excluded common genetic variants between the Heilmann-Heimbach et al. and Hagenaars et al.**

| Genetic variants |
| --- |
| rs12083887 |
| rs11684254 |
| rs12203592 |
| rs62060349 |

**S3C Table: Excluded genetic variants as no proxy was found**

| Genetic variants | Study name |
| --- | --- |
| rs34061913 | Heilmann-Heimbach et al. |
| rs144578168 | Heilmann-Heimbach et al. |
| rs201655553 | Heilmann-Heimbach et al. |
| rs201796065 | Hagenaar et al. |
| rs145126703 | Hagenaar et al. |
| rs199978639 | Hagenaar et al. |
| rs112949019 | Hagenaar et al. |

**S3D Table: Excluded genetic variants as R^2^≤0.8 with the proxies**

| Genetic variants |
| --- |
| rs199961668 |
| rs111668293 |
| rs150489034 |
| rs35534179 |
| rs4976028 |

**S3E Table: Excluded 88 genetic variants with minor allele frequency < 0.05**

| Genetic variants | Genetic variants | Genetic variants | Genetic variants |
| --- | --- | --- | --- |
| rs62390189 | rs74895226 | rs146112277 | rs76067940 |
| rs150909339 | rs77781418 | rs143755874 | rs145562694 |
| rs2294736 | rs77775907 | rs138876904 | rs79206101 |
| rs139092879 | rs9282858 | rs147154263 | rs77767830 |
| rs1543354 | rs191212334 | rs73213393 | rs118013985 |
| rs141173114 | rs72787520 | rs147670940 | rs77096234 |
| rs142020459 | rs72787535 | rs145867342 | rs183207557 |
| rs17035390 | rs72787555 | rs112069404 | rs55908337 |
| rs2242288 | rs112550936 | rs144393202 | rs75520281 |
| rs72809169 | rs143942024 | rs17216820 | rs115182912 |
| rs117584427 | rs140843301 | rs146636673 | rs17626412 |
| rs77527369 | rs141433484 | rs73227823 | rs13405699 |
| rs73071393 | rs140707533 | rs62604342 | rs16862069 |
| rs6113382 | rs185597083 | rs113222435 | rs71421546 |
| rs78297031 | rs143212632 | rs148652266 | rs71421553 |
| rs12625546 | rs139470886 | rs73212804 | rs34769088 |
| rs143916866 | rs147829649 | rs17302236 | rs6755476 |
| rs188468174 | rs73209413 | rs73212868 | rs116315504 |
| rs186127900 | rs5989004 | rs140488081 | rs12123537 |
| rs79472072 | rs150535953 | rs117463770 | rs78448052 |
| rs78321654 | rs140914450 | rs141577316 | rs192913879 |
| rs11049231 | rs111810219 | rs73837363 | rs148541866 |
